# Incidence of relapsed/refractory diffuse large B-cell lymphoma (DLBCL) including CNS relapse in a population-based cohort of 4243 patients in Sweden

**DOI:** 10.1038/s41408-020-00403-1

**Published:** 2021-01-07

**Authors:** Sara Harrysson, Sandra Eloranta, Sara Ekberg, Gunilla Enblad, Mats Jerkeman, Bjorn E. Wahlin, Per-Ola Andersson, Karin E. Smedby

**Affiliations:** 1 Division of Clinical Epidemiology, Department of Medicine Solna, Karolinska Institutet, Stockholm, Sweden; 2 Department of Hematology, Karolinska University Hospital, Solna, Sweden; 3 Department of Immunology, Genetics, and Pathology, Uppsala University, Uppsala, Sweden; 4 Department of Oncology, Lund University, Lund, Sweden; 5 Division of Hematology, Department of Medicine Huddinge, Karolinska Institutet, Stockholm, Sweden; 6 Department of Hematology, South Älvsborg Hospital, Borås, and Sahlgrenska Academy, Gothenburg University Sweden, Gothenburg, Sweden

**Keywords:** Epidemiology, Cancer epidemiology, B-cell lymphoma

## Abstract

We performed a national population-based study of all patients diagnosed with diffuse large B-cell lymphoma (DLBCL) in Sweden in 2007–2014 to assess treatment intent and risk of relapsed/refractory disease, including central nervous system (CNS) relapse, in the presence of competing risks. Overall, 84% of patients started treatment with curative intent (anthracycline-based) (*n* = 3550, median age 69 years), whereas 14% did not (*n* = 594, median age 84 years) (for 2% the intent was uncertain). Patients treated with curative intent had a 5-year OS of 65.3% (95% CI: 63.7–66.9). The median OS among non-curatively treated patients was 2.9 months. The 5-year cumulative incidence of relapsed/refractory disease in curative patients was 23.1% (95% CI: 21.7–24.6, *n* = 847). The 2-year cumulative incidence of CNS relapse was 3.0% (95% CI: 2.5–3.6, *n* = 118) overall, and 8.0% (95% CI: 6.0–10.6, *n* = 48) among patients with high CNS-IPI (4–6), when considering other relapse locations and death as competing events. The incidence of relapsed/refractory DLBCL overall and in the CNS was lower than in previous reports, still one in seven patients was not considered fit enough to start standard immunochemotherapy at diagnosis. These results are important for quantification of groups of DLBCL patients with poor prognosis requiring completely different types of interventions.

## Introduction

Diffuse large B-cell lymphoma (DLBCL) is the most common subtype of lymphoma and has an aggressive clinical course^1^. Outcomes have improved in the last decades with the addition of rituximab to standard anthracycline-based chemotherapy^2–6^. A proportion of patients, however, still experience primary refractory disease or relapse, with a dramatic worsening of the prognosis, especially if the relapse occurs within 1 year of diagnosis, or if located in the central nervous system (CNS)^7,8^.

Known clinical risk factors for relapse include advanced stage, high age, elevated serum lactate dehydrogenase (LDH), poor performance status and involvement of more than one extranodal site, as summarized in the widely used International prognostic index (IPI) score^9^. To estimate the risk of central nervous system (CNS) relapse, the CNS-IPI was developed based on IPI with the addition of kidney and/or adrenal gland involvement^10^.

Now 20 years ago, gene-expression profiling techniques were used to identify biological subtypes of DLBCL by cell of origin, the germinal center B-cell (GCB) and activated B-cell (ABC) subtypes, where the ABC subtype has been associated with worse prognosis^11–13^. More recently, four or five different genetic signatures have been proposed that better describe the biological pathways of disease development, with prognostic and predictive implications^14–16^. Although the impact of these findings in clinical routine is yet to be defined, they provide important guidance for the ongoing development and use of new targeted therapies.

In spite of large ongoing efforts to improve risk prediction and treatment of relapsed/refractory disease in DLBCL, there is still uncertainty about the true proportion of patients who experience relapsed/refractory disease in unselected cohorts, and figures ranging from 30 to 40% are often cited^17–20^. A correct estimation of the incidence can help to quantify patient groups eligible for new treatments and other interventions aiming to improve outcome.

Therefore, we set out to perform a national population-based study to provide real-world absolute risk estimates of relapsed/refractory disease in DLBCL in the rituximab era, using register data with validation of treatment response and relapse via medical charts review.

## Patients and methods

### Study design and study population

We used the Swedish Lymphoma Register (SLR) to identify all patients with a primary diagnosis of DLBCL in Sweden from January 2007 through December 2014. The SLR is a national quality-of-care register to which physicians report information on clinical characteristics and primary treatment for all newly diagnosed lymphoma patients in Sweden. The coverage is high; 95% compared to the mandatory National Cancer register. The register is regularly linked to the Swedish population register for information on vital status and dates of death. Relapses have been registered in the SLR since 2010 with incomplete coverage. For the purposes of this study, medical charts of all registered DLBCL patients 2007–2014 were, therefore, reviewed to complement and validate the information regarding treatment intent, response to first-line treatment and occurrence of relapse. Specially trained research nurses and/or medical staff at local hospitals performed the chart review and updated the register within the regular register reporting system. Additional informed consent from the patients to complete register data was not required. However, a study-specific informed consent was obtained from live patients with relapsed/refractory disease to ascertain location of relapse.

We identified 4805 patients with a primary diagnosis of DLBCL in the SLR. We excluded two patients diagnosed at autopsy and 112 patients (2%) due to inability to access their medical charts. Following medical chart review, we further excluded patients with primary CNS lymphoma (*n* = 240, 5%), primary mediastinal lymphoma (*n* = 104, 2%), discordant/transformed lymphoma (*n* = 61, 1%), and 43 patients (0.9%) with other lymphoma diagnoses. The final cohort consisted of 4243 DLBCL patients (Online Fig. 1).

Among patients with primary refractory disease or relapse (*n* = 847), 15 living patients did not consent to medical chart review and in 27 patients information about relapse location was missing. These 42 patients (5.2% of all relapses) were excluded from analyses regarding CNS relapse.

### Clinical characteristics and primary treatment

Clinical characteristics including ECOG performance status, Ann Arbor stage, LDH, involved extranodal sites and administered primary treatment regimens were obtained from the SLR. During the study period, the Swedish National DLBCL treatment guidelines recommended R-CHOP (rituximab, cyclophosphamide, doxorubicin, vincristine, prednisone) 14 or 21 as first-line treatment for most patients. Younger patients (≤65 years) with age-adjusted IPI 2–3 were recommended R-CHOEP-14 (R-CHOP + etoposide), and consolidation with high-dose methotrexate/high-dose cytarabine (as in the Nordic CRY-04-trial)^21^. Intrathecal prophylaxis was recommended only to patients with testicular involvement, but was also used for, e.g., sinonasal involvement or advanced disease in general.

In the study, curative intent was defined as having started treatment with an anthracycline-containing regimen (mostly R-CHOP). R-CEOP (etoposide instead of doxorubicin) and more intense treatments including Hyper-CVAD (fractionated cyclophosphamide, doxorubicin, vincristine, dexamethasone alternated with high-dose methotrexate and cytarabine), BFM 04 (the Berlin-Frankfurt-Münster protocol) and GMALL-B-ALL/NHL 2002 (a German multicenter B-ALL and high-grade B-cell lymphoma protocol) were also considered curative.

### Definition of outcome

The main outcome was primary refractory disease or high-grade relapse among patients treated with curative intent. Primary refractory disease was defined as having no response to primary treatment (i.e., stable or progressive disease, SD/PD). Other outcomes of interest were overall survival (OS), defined as time from diagnosis to death of any cause, and progression-free survival (PFS), defined as time from diagnosis to refractory disease, high-grade relapse or death of any cause^22^. CNS progression/relapse was based on typical MRI/CT findings or cerebrospinal fluid analysis with cytology and/or flow cytometry.

The study was approved by the regional ethics committee in Stockholm (Dnr 2015/2028-31/2).

### Statistical analysis

The patients were followed from diagnosis until the date of the event (refractory disease or high-grade relapse), death or October 31 2017 (starting date for the medical chart review), whichever came first. OS was estimated using the Kaplan–Meier method among all patients (non-curatively and curatively treated) whereas estimation of PFS was limited to curatively treated patients. The cumulative incidence of relapsed/refractory disease was estimated non-parametrically in the presence of the competing risk of death, and stratified by age at diagnosis (>/≤70) and IPI (0–1/2/3/4–5). Separate analyses were performed among younger patients (<60) with aaIPI 0–1 or 2–3 and among patients who received ≥3 anthracycline-containing chemotherapy cycles.

The cumulative incidence of CNS progression/relapse was estimated overall, by age at diagnosis (>/≤70) and by CNS IPI (0–1/2–3/4–6) among curatively treated patients with no CNS involvement at diagnosis and included death and non-CNS progression/relapse as competing events. The corresponding net probabilities of CNS progression/relapse (i.e., in the absence of competing risks) were also estimated using the Kaplan–Meier method.

Cause-specific Cox models where relapses were classified as events and deaths as censored observations were used to estimate the association between clinical characteristics and risk of progression/relapse overall and in the CNS. The multivariable models were adjusted for age at diagnosis, sex, performance status, stage, LDH, number of extranodal sites and IPI/CNS IPI. The proportional hazards assumption was tested using the Schoenfeld residuals.

All statistical analyses were performed using STATA version 14.1 (Stata Corp., College Station, TX, USA) and the code used to generate the results is stored in the electronic notebook; KI ELN at Karolinska Institutet.

## Results

### Patient characteristics, OS, and PFS

**Fig. 1:**
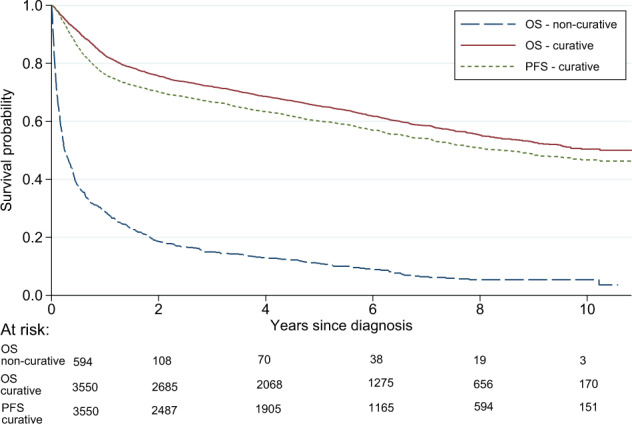
Overall survival and progression-free survival. Overall survival (OS) among curatively (*n* = 3550) and non-curatively (*n* = 594) treated diffuse large B-cell lymphoma (DLBCL) patients diagnosed 2007–2014 in Sweden, and progression-free survival (PFS) among curatively treated patients.

Of the 4243 patients in the final cohort, 3550 (84%) were treated with curative intent (Table 1) and 594 (14%) did not receive curative treatment. Data were missing regarding first-line treatment in 99 patients, 2.3%. Median age at diagnosis was 71 years overall (range 18–105), 69 years in the curative group and 84 years in the non-curative (Table 1). Five-year OS for patients treated with curative intent was 65.3% (95% CI: 63.7–66.9). Two-year PFS was 70.1% (95% CI: 68.5–71.6) and 5-year PFS was 60.1% (95% CI: 58.4–61.7). Patients treated non-curatively had a median OS of 2.9 months and a 5-year OS of 11.0% (95% CI: 8.6–13.8) (Fig. 1). Among the non-curatively treated patients, 134 (3.2% of the whole cohort) received low-intensity intravenous regimens (e.g., bendamustine or CVP (cyclophosphamide, vincristine, prednisone)), 117 (2.8%) radiotherapy alone, and 315 patients (7.4%) had no active treatment (online Table 1).

**Table 1:** Clinical characteristics among all patients diagnosed with diffuse large B-cell lymphoma (DLBCL) 2007–2014 in Sweden, and in curatively and non-curatively treated patients separately.

	All DLBCL patients *n* (%)	Curative^a^ *n* (%)	Non-curative *n* (%)
	*N* = 4243 (100)	*N* = 3550 (84)	*N* = 594 (14)
Age at diagnosis			
Median (range)	71 (18–105)	69 (18-99)	84 (38-105)
<50	438 (10.3)	423 (11.9)	10 (1.7)
50–59	455 (10.7)	440 (12.4)	12 (2.0)
60–69	1064 (25.1)	1014 (28.6)	36 (6.1)
70–79	1199 (28.3)	1059 (29.8)	113 (19.0)
80+	1087 (25.6)	614 (17.3)	423 (71.2)
Sex			
Female	1894 (44.6)	1528 (43.0)	320 (53.9)
Male	2349 (55.4)	2022 (57.0)	274 (46.1)
Ann Arbor stage			
I	856 (20.2)	724 (20.4)	123 (20.7)
II	828 (19.5)	732 (20.6)	87 (14.7)
III	829 (19.5)	740 (20.9)	74 (12.5)
IV	1523 (35.9)	1287 (36.3)	194 (32.7)
Unknown	207 (4.9)	67 (1.9)	116 (19.5)
LDH			
Normal	1620 (38.2)	1404 (39.6)	203 (34.2)
Elevated	2421 (57.1)	2081 (58.6)	288 (48.5)
Unknown	202 (4.8)	65 (1.8)	103 (17.3)
ECOG PS			
0	1776 (41.9)	1682 (47.4)	81 (13.6)
1	1377 (32.5)	1199 (33.8)	162 (27.3)
2	446 (10.5)	346 (9.8)	90 (15.2)
3	378 (8.9)	224 (6.3)	136 (22.9)
4	190 (4.5)	74 (2.1)	92 (15.5)
Unknown	76 (1.8)	25 (0.7)	33 (5.6)
Extranodal sites			
0	2231 (52.6)	1858 (52.3)	319 (53.7)
1	1367 (32.2)	1148 (32.3)	194 (32.7)
>1	645 (15.2)	544 (15.3)	81 (13.6)

*LDH* lactate dehydrogenase.

^a^Curative treatment was defined as having started treatment with anthracycline-containing chemotherapy. 99 patients (2%) had missing data regarding primary treatment.

Curatively treated patients received mostly R-CHOP (90.9%) followed by R-CHOEP (5.8%) and R-CEOP (1.6%) (Table 2). Among all curatively treated patients 15.7% (*n* = 558) received CNS prophylaxis, of whom 8.7% (*n* = 307) were given intrathecal prophylaxis only, and 7.1% (*n* = 251) systemic prophylaxis with high-dose methotrexate and/or cytarabine (sometimes in combination with intrathecal methotrexate) (Table 2). Thirty-six (0.8%) patients presented with CNS involvement at diagnosis and 30 of them where treated with curative intent.

**Table 2:** International prognostic index (IPI), CNS-IPI, first-line treatment and treatment response in all DLBCL patients treated with curative intent, and patients who experienced relapsed/refractory disease during follow-up (CNS progression/relapse presented separately).

	All curatively treated patients (*n* = 3550)	Relapsed/refractory disease (*n* = 847)	Cohort assessed for CNS relapse^a^ (*n* = 3478)	CNS relapse (*n* = 118)
IPI				
0	244 (6.9)	13 (1.5)	243 (7.0)	0 (0.0)
1	786 (22.1)	121 (14.3)	779 (22.4)	8 (6.8)
2	961 (27.1)	213 (25.2)	946 (27.2)	27 (22.9)
3	857 (24.1)	264 (31.2)	844 (24.3)	38 (32.2)
4	454 (12.8)	159 (18.8)	433 (12.5)	34 (28.8)
5	105 (3.0)	41 (4.8)	95 (2.7)	10 (8.5)
Unknown	143 (4.0)	36 (4.3)	138 (4.0)	1 (0.9)
CNS-IPI^a^				
0	244 (6.9)	13 (1.6)	243 (7.0)	0 (0.0)
1	784 (22.3)	120 (14.4)	777 (22.3)	8 (6.8)
2	951 (27.0)	211 (25.3)	939 (27.0)	25 (21.2)
3	846 (24.0)	260 (31.2)	836 (24.0)	36 (30.5)
4	426 (12.1)	145 (17.4)	418 (12.0)	32 (27.1)
5	108 (3.1)	38 (4.6)	106 (3.1)	11 (9.3)
6	21 (0.6)	12 (1.4)	21 (0.6)	5 (4.2)
Unknown	140 (4.0)	35 (4.2)	138 (4.0)	1 (0.9)
Primary treatment				
R-CHOP	3226 (90.9)	743 (87.7)	3182 (91.5)	100 (84.7)
R-CHOEP	205 (5.8)	67 (7.9)	190 (5.5)	13 (11.0)
R-DA-EPOCH	9 (0.3)	2 (0.2)	9 (0.3)	0 (0.0)
R-CEOP	55 (1.6)	15 (1.8)	53 (1.5)	1 (0.9)
HyperCVAD/BFM/GMALL	40 (1.1)	13 (1.5)	30 (0.9)	2 (1.7)
Other potentially curative	15 (0.4)	7 (0.8)	14 (0.4)	2 (1.7)
No. of cycles received				
1–2	238 (6.7)	46 (5.4)	225 (6.5)	4 (3.4)
3–5	439 (12.4)	121 (14.3)	431 (12.4)	8 (6.8)
6	2644 (74.5)	610 (72.0)	2601 (74.8)	101 (85.6)
>6	216 (6.1)	62 (7.3)	209 (6.0)	3 (2.5)
Unknown	13 (0.4)	8 (1.0)	12 (0.4)	2 (1.7)
CNS prophylaxis^b^				
Intrathecal	307 (8.7)	86 (10.3)	305 (8.7)	17 (14.4)
Systemic	95 (2.7)	27 (3.2)	93 (2.7)	6 (5.1)
Both	156 (4.4)	47 (5.6)	153 (4.4)	10 (8.5)
No CNS prophylaxis	2962 (84.1)	674 (80.8)	2927 (84.1)	85 (72.0)
Radiotherapy				
Yes	366 (89.6)	82 (9.7)	358 (10.3)	10 (8.5)
No	3180 (10.3)	762 (90.0)	3116 (89.6)	107 (90.7)
Unknown	4 (0.1)	3 (0.3)	4 (0.1)	1 (0.8)
Outcome of primary treatment				
CR	2618 (73.8)	419 (49.5)	2585 (74.3)	65 (55.1)
PR	377 (10.6)	137 (16.2)	364 (10.5)	18 (15.3)
SD	62 (1.7)	62 (7.3)	59 (1.7)	3 (2.5)
PD	203 (5.7)	203 (23.9)	192 (5.5)	29 (24.6)
Not formally evaluated^c^	290 (8.2)	26 (3.1)	278 (8.0)	3 (2.5)

^a^Includes all curatively treated patients without CNS involvement at diagnosis, who gave consent to medical chart review and where information regarding relapse location was available.

^b^Patients with CNS involvement at diagnosis were excluded (*n* = 30).

^c^Includes patients judged to have a clinical response, i.e., not stable or progressive disease (SD/PD), but who never performed radiographic evaluation, mainly due to early interruption of treatment due to toxicity (*n* = 234) or for unknown reasons (*n* = 56).

### Outcome of primary treatment and incidence of relapse

Among curatively treated patients, the overall response rate was 84.4% (CR 73.8%, PR 10.6%). In total, 7.5% (*n* = 265) had SD (1.8%) or PD (5.7%) as best response (Table 2). A proportion of 8.2% (*n* = 290) of the patients were judged to have a clinical response (i.e., not SD/PD) but were not formally evaluated; 6.6% (*n* = 234) because of early treatment interruption due to toxicity and 1.9% (*n* = 56) for unknown reasons. Overall, 32% of patients were evaluated for response with PET-CT (the proportion was similar across the study period).

**Fig. 2:**
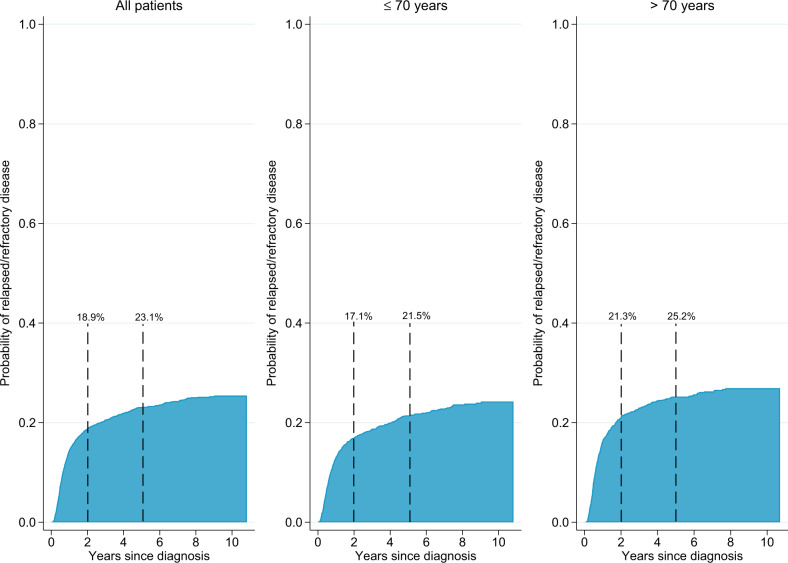
Incidence of relapsed/refractory disease. Cumulative incidence of relapsed/refractory disease at any site among curatively treated diffuse large B-cell lymphoma (DLBCL) patients overall (*n* = 3550, 847 events), and by age at diagnosis (>/≤ 70 years) (incidence assessed in the presence of the competing risk of death*). Asterisk indicates Not depicted.

In total, 847 patients were primary refractory or experienced high-grade relapse during a median follow-up of 4.3 years (Table 2). The 2-year incidence of relapsed/refractory disease was 18.9% (95% CI: 17.7–20.2) and the 5-year incidence was 23.1% (95% CI: 21.7–24.6) in the presence of the competing risk of death (Fig. 2). The incidence was identical when restricting the analysis to patients who received ≥3 cycles of chemotherapy (*n* = 3299, 793 events) (Online Fig. 2). Among patients with relapsed/refractory disease, 62% (*n* = 521) manifested within the first year, and 79% (*n* = 671) within 2 years of diagnosis (Online Fig. 3). Thirty-nine patients (4.6%) had a relapse after 5 years or more.

**Fig. 3:**
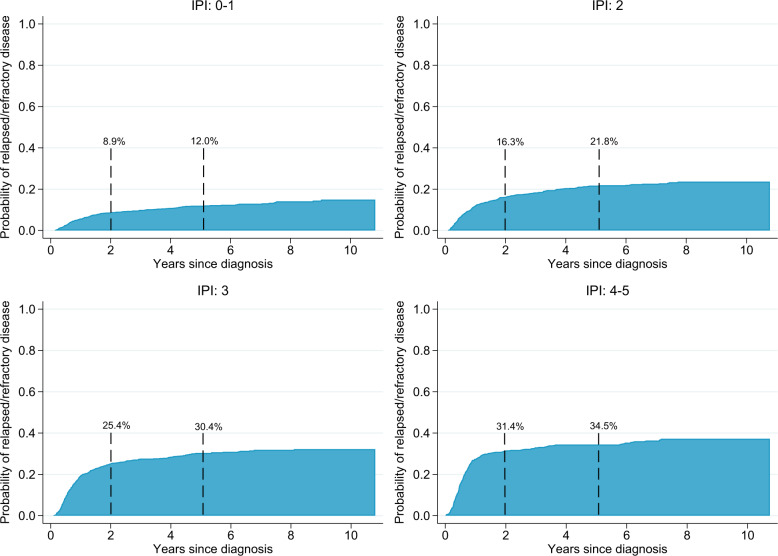
Incidence of relapsed/refractory disease by IPI. Cumulative incidence of relapsed/refractory disease at any site among curatively treated diffuse large B-cell lymphoma (DLBCL) patients by IPI (0–1, 2, 3, 4–5) (incidence assessed in the presence of the competing risk of death*). Asterisk indicates not depicted.

The incidence of relapsed/refractory disease was slightly higher among patients aged >70 years (2 years: 21.3%, 95% CI: 19.3–23.3, 5 years: 25.2%, 95% CI: 23.1–27.4) than ≤70 years (2 years: 17.1%, 95% CI: 15.4–18.8, 5 years: 21.5%, 95% CI: 19.7–23.3) (Fig. 2). When estimated separately for patients with IPI 0–1, 2, 3 and 4–5, the 2-year incidence was 8.9%, 16.3%, 25.4%, and 31.4%, respectively (at 5 years 12.0%, 21.8%, 30.4%, and 34.5%) (Fig. 3). Among younger patients (<60) with aaIPI 0–1 the 2-year incidence was 8.7%, 95% CI: 6.5–11.3 (at 5 years 11.7%, 95% CI: 9.0–14.7) and with aaIPI 2–3 it was 24.6%, 95% CI: 20.5–28.9 (at 5 years 31.0%, 95% CI: 26.4–35.6) (Online Fig. 4). The risk of relapsed/refractory disease was further confirmed to be associated with the known clinical risk factors included in the IPI in a multivariable Cox regression analysis (Table 3). Primary involvement of the CNS (HR = 2.0, 95% CI: 1.1–3.6), bone marrow (HR = 1.6, 95% CI: 1.3–1.9), testis (HR = 1.5, 95% CI: 1.0–2.1), kidney/adrenal glands (HR = 1.4, 95% CI: 1.0–2.0) or lung (HR = 1.3, 95% CI: 1.0–1.7) were also associated with increased risk of relapse.

**Table 3:** Clinical characteristics and relative risk (hazard ratios (HR) with 95% confidence intervals (CI)) of relapsed/refractory disease at any site or in the CNS among curatively treated diffuse large B-cell lymphoma (DLBCL) patients diagnosed 2007–2014 in Sweden.

	Risk of any relapse^a^ (*N* = 3407)			Risk of CNS relapse^a^ (*N* = 3340)		
Variables	Events *N* (%)	HR (95% CI) Unadjusted	HR (95% CI) Adjusted^b^	Events *N* (%)	HR (95% CI) Unadjusted	HR (95% CI) Adjusted^b^
Age, years						
<50	67 (8.3)	1.0 (ref)	1.0 (ref)	8 (6.8)	1.0 (ref)	1.0 (ref)
50–59	102 (12.6)	1.5 (1.1–2.1)	1.5 (1.1–2.0)	15 (12.8)	1.9 (0.8–4.4)	1.7 (0.7–4.1)
60–69	241 (29.7)	1.7 (1.3–2.2)	1.5 (1.1–1.9)	46 (39.3)	2.2 (1.0–4.6)	
70–79	252 (31.1)	1.8 (1.4–2.4)	1.7 (1.3–2.2)	36 (30.8)	2.1 (1.0–4.4)	1.8 (0.8-3.89
80+	149 (18.4)	2.4 (1.8–3.2)	2.3 (1.7–3.0)	12 (10.3)	1.5 (0.6–3.6)	1.3 (0.5–3.2)
Sex						
Female	317 (39.1)	1.0 (ref)	1.0 (ref)	48 (41.0)	1.0 (ref)	1.0 (ref)
Male	494 (60.9)	1.2 (1.1–1.4)	1.3 (1.1–1.5)	69 (59.0)	1.1 (0.8–1.6)	1.2 (0.8–1.8)
ECOG PS						
0	313 (38.6)	1.0 (ref)	1.0 (ref)	38 (32.5)	1.0 (ref)	1.0 (ref)
1	299 (36.9)	1.6 (1.4–1.9)	1.2 (1.0–1.4)	38 (32.5)	1.6 (1.0–2.6)	1.1 (0.7–1.8)
2	106 (13.1)	2.5 (2.0–3.1)	1.6 (1.2–2.0)	23 (19.7)	4.3 (2.6–7.3)	2.5 (1.5–4.3)
3	67 (8.3)	2.7 (2.1–3.5)	1.6 (1.2–2.0)	11 (9.4)	3.6 (1.9–7.1)	1.8 (0.9–3.6)
4	26 (3.2)	4.7 (3.2–7.0)	2.6 (1.7–4.0)	7 (6.0)	10.0 (4.4–22.4)	4.4 (1.9–10.1)
Ann Arbor stage						
I	81 (10.0)	1.0 (ref)	1.0 (ref)	8 (6.8)	1.0 (ref)	1.0 (ref)
II	134 (16.5)	1.7 (1.3–2.3)	1.4 (1.1–1.9)	11 (9.4)	1.4 (0.6–3.6)	1.0 (0.4–2.6)
III	190 (23.4)	2.6 (2.0–3.4)	1.9 (1.5–2.5)	22 (18.8)	3.0 (1.3–6.8)	1.8 (0.8–4.1)
IV	406 (50.1)	3.6 (2.9–4.6)	2.4 (1.8–3.0)	76 (65.0)	6.8 (3.3–14.1)	3.3 (1.5–7.2)
LDH						
Normal	211 (26.0)	1.0 (ref)	1.0 (ref)	18 (15.4)	1.0 (ref)	1.00 (ref)
Elevated	600 (74.0)	2.3 (2.0–2.7)	1.8 (1.5–2.1)	99 (84.6)	4.4 (2.7–7.3)	2.8 (1.6–4.7)
Extranodal sites						
0	355 (43.8)	1.0 (ref)	1.0 (ref)	36 (30.8)	1.0 (ref)	1.0 (ref)
1	293 (36.1)	1.4 (1.2–1.6)	1.1 (0.9–1.3)	47 (40.2)	2.2 (1.4–3.4)	1.5 (0.9–2.4)
2	111 (13.7)	1.8 (1.4–2.2)	1.1 (0.9–1.4)	19 (16.2)	3.0 (1.7–5.2)	1.6 (0.9–2.8)
3 or more	52 (6.4)	2.2 (1.6–2.9)	1.3 (0.9–1.7)	15 (12.8)	6.6 (3.6–12.1)	3.0 (1.6–5.6)
IPI						
0-1	134 (16.5)	1.0 (ref)	1.0 (ref)			
2	213 (26.3)	1.9 (1.5–2.4)	1.9 (1.5–2.4)			
3	264 (32.6)	3.0 (2.4–3.7)	3.1 (2.5–3.8)			
4	159 (19.6)	4.1 (3.3–5.2)	4.2 (3.3–5.2)			
5	41 (5.1)	5.5 (3.9–7.8)	5.6 (3.9–7.9)			
CNS-IPI						
0–1				8 (6.9)	1.0 (ref)	1.0 (ref)
2–3				61 (52.1)	5.0 (2.4–10.5)	5.1 (2.4–10.6)
4–6				48 (41.0)	16.5 (7.8–34.9)	16.7 (7.9–35.3)
Extranodal involvement at diagnosis						
Bone	72 (8.9)	1.1 (0.8–1.4)	0.8 (0.6–1.0)	14 (12.0)	1.5 (0.9–2.7)	0.9 (0.5–1.7)
Bone marrow	133 (16.4)	2.4 (2.0–2.9)	1.6 (1.3–1.9)	31 (26.5)	4.3 (2.9–6.5)	2.3 (1.5–3.5)
CNS	12 (1.5)	3.1 (1.7–5.4)	2.0 (1.1–3.6)	-	-	-
Colon	23 (2.8)	0.9 (0.6–1.4)	0.7 (0.5–1.1)	3 (2.6)	0.9 (0.3–2.7)	0.7 (0.2–2.1)
Kidney/adrenal	36 (4.4)	2.2 (1.5–3.0)	1.4 (1.0–2.0)	17 (14.5)	8.1 (4.8–13.5)	4.1 (2.4–7.0)
Liver	41 (5.1)	1.1 (0.8–1.5)	0.7 (0.5–1.0)	6 (5.1)	1.1 (0.5–2.6)	0.5 (0.2–1.2)
Lung	65 (8.0)	1.8 (1.4–2.3)	1.3 (1.0–1.7)	13 (11.1)	2.5 (1.4-4.4)	1.6 (0.9–2.9)
Muscle	18 (2.2)	1.1 (0.7–1.7)	0.8 (0.5–1.2)	5 (4.3)	2.2 (0.9–5.4)	1.4 (0.5–3.4)
Ovaries/uterus	8 (1.0)	1.3 (0.6–2.5)	1.3 (0.7–2.7)	2 (1.7)	2.2 (0.6–9.0)	2.2 (0.5–9.0)
Small intestine	30 (3.7)	0.9 (0.6–1.2)	0.8 (0.6–1.2)	5 (4.3)	1.0 (0.4–2.5)	0.9 (0.4–2.2)
Testis	31 (3.8)	1.5 (1.0–2.1)	1.5 (1.0–2.1)	3 (2.6)	1.0 (0.3–3.1)	1.1 (0.3–3.4)
Ventricle	29 (3.6)	0.7 (0.5–1.0)	0.6 (0.4–0.9)	7 (6.0)	1.1 (0.5–2.4)	1.0 (0.5–2.1)

^a^143 patients with missing IPI were excluded from analysis of risk of any relapse and 138 patients with missing CNS-IPI were excluded from the analysis of risk of CNS-relapse.

^b^Age, sex, ECOG performance status, Ann Arbor stage and LDH were included in one multivariable model. Extranodal involvement was adjusted for age, sex, ECOG performance status, LDH and stage. IPI and CNS-IPI were adjusted for sex.

### CNS relapse

Progression/relapse in the CNS occurred in 118 patients at a median of 7 months after diagnosis (Online Fig. 3) and of these relapses, 78 (66%) were limited to the CNS only. Most patients who experienced CNS relapse (*n* = 69, 58%) had an initial CNS IPI score of 0–3, and approximately one in four patients (*n* = 33, 28%) had received initial CNS prophylaxis (Table 2).

**Fig. 4:**
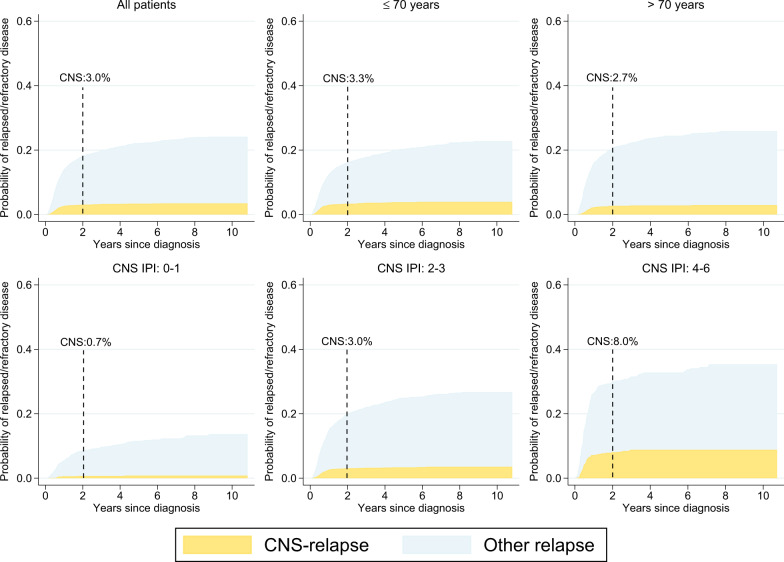
Incidence of CNS relapse. Cumulative incidence of CNS relapse among curatively treated diffuse large B-cell lymphoma (DLBCL) patients overall (for whom detailed medical records were available, *n* = 3478, 118 events), and by age at diagnosis and CNS-IPI risk group, respectively (incidence assessed in the presence of the competing risks of relapse without CNS involvement and death*). Asterisk indicates not depicted.

The 2-year incidence of CNS relapse was 3.0% (95% CI: 2.5–3.6) with little variation by age (>70: 2.7% (95% CI: 1.9–3.5); ≤70: 3.3% (95% CI: 2.6–4.2)) (Fig. 4). By CNS-IPI 0–1, 2–3 and 4–6 the 2-year incidences of CNS relapse were 0.7%, 3.0%, and 8.0% respectively (Fig. 4), which rose to 11.8% if restricting to CNS-IPI 5-6 (*n* = 127, 16 events, Online Fig. 5). The corresponding 2-year risk estimates in the absence of competing risks (i.e., using the Kaplan–Meier method) were higher (Online Fig. 6). In a multivariable regression model, the risk of CNS relapse was confirmed to be strongly associated with the CNS-IPI score and its included variables with the exception of age 70 years and above (Table 3). Number of extranodal sites (≥3 sites HR = 3.0, 95% CI:1.6–5.6) and involvement of the kidney/adrenal glands (HR = 4.1, 95% CI: 2.4–7.0) and bone marrow (HR = 2.3, 95% CI: 1.5–3.5) were also independently associated with risk.

## Discussion

In this large nationwide population-based study, we demonstrate that the proportion of DLBCL patients overall that experience progression or relapse is only 18.9% at 2 years and 23.1% at 5 years, thus considerably lower than generally stated^17–20^. Also the incidence of CNS relapse was low; 3.0% in the whole cohort and 8.0% among high-risk patients (CNS IPI 4–6). We believe these real-world benchmark figures are important for patients as well as physicians and policy makers to help estimate patient numbers eligible for new treatments in the era of targeted therapies for relapsed/refractory disease. At the same time, we identified a relatively large proportion (14%) of mostly older patients (median age 84 years) who were not considered fit enough to even start intensive treatment with anthracycline-containing regimens, and thus are in need of alternate primary interventions.

With the addition of rituximab to standard therapy with CHOP, the outcome for DLBCL patients has improved in the last decades. For instance, the RICOVER-60 trial reported a 3-year event-free survival of 66.5% after six cycles of R-CHOP-14 in patients aged 61–80 years^4^. In the GOYA-trial where CHOP + obinutuzumab was compared with R-CHOP for DLBCL patients >18 years, an estimated 3-year PFS of 67% was reported in the R-CHOP-arm^23^. A large register-based study comparing outcomes for advanced-stage patients treated before and after the implementation of rituximab in the province of British Columbia, reported a 2-year PFS of 69% in R-CHOP treated patients^6^. These studies report outcomes that are similar to ours in terms of PFS (with a 2-year PFS of 70% and a 5-year PFS of 60% in our study). However, since PFS reflects failure rates including both relapsed/refractory disease and deaths due to other causes, the PFS figure does not necessarily give a correct estimate of patients with insufficient response to primary treatment, but also incorporates patients who died for reasons related to toxicity or other unrelated causes. Also, when estimating the cumulative incidence of relapsed/refractory disease, we took the competing risk of death from other causes into account, which is advisable in elderly populations.

We further investigated incidence of CNS relapse among curatively treated patients, and observed a relatively low rate of CNS relapse. This is in line with some previous reports of a low incidence of CNS relapse in the rituximab era^8,24^. However, to our knowledge, no previous study has presented incidence of CNS relapse in the real-world accounting for competing risks of death or other non-CNS relapses. In the pivotal study by Schmitz et al.^10^ introducing the CNS-IPI score, net incidence of CNS relapse was used to confirm the ability of the CNS-IPI to single out the patients with the highest risk of CNS disease. In that report, patients with high CNS IPI^4–6^ had a net probability of CNS relapse of 10–12%, whereas the incidence in this group in our study was 8%.

CNS relapse is a clinically dreaded event that is desirable to prevent, although the optimal prophylactic strategy remains debated and there are no randomized trials to guide primary prophylactic interventions. In a recent review, it was concluded that the use of intrathecal chemotherapy prophylaxis seem to have no clear protective effect^25^. With improvements in the outcomes of primary CNS lymphoma in recent years, using treatment protocols based on the CNS penetrating drugs methotrexate and cytarabine, it has become more common to adopt a similar first-line treatment strategy to prevent CNS relapse^21^. This is however, a strategy that takes a lot of medical resources and that also increases the risk of toxicity for the individual patient. For these reasons, it is paramount to identify the patients who best benefit from such therapy. Of note, more than half (58%) of the patients who eventually experienced CNS relapse in our study had in fact low or intermediate CNS-IPI scores of 0–3, and would with current treatment strategies, used at most centers, not receive CNS prophylaxis. This highlights the need for even better clinical and molecular tools to identify the patients who most benefit from intensified treatment strategies with CNS penetrating drugs.

The patients who could not start curative treatment at diagnosis represent another group of mostly older patients where better treatment options are urgently needed. Perhaps a larger proportion would be candidates for R-CHOP if pre-phase treatment with steroids and vincristine was used as suggested in a few studies^26,27^. It is possible that a few patients received pre-phase treatment in our study, although this was not recorded, however, this was not an established treatment schedule in the National Swedish Guidelines during the study period. Pre-planned dose adjustments could also be an alternative for the elderly and/or frail patients^28^, maybe with the addition of newer drugs such as polatuzumab-vedotin^29^.

The strengths of our study include the nationwide setting and the use of clinical detail through both a clinical register and validation of treatment response and relapse through medical record review. Weaknesses include the retrospective design, which means that we could not incorporate molecular classifications of cell of origin or genetic aberrations in *MYC*, *BCL-2*, or *BCL-6* since they were not yet in use in clinical routine during most of the study period. However, although relapse rates will vary by tumor characteristics and DLBCL subtypes, overall estimates of relapsed/refractory disease are still valid. Also, some of the treatment guidelines, e.g., regarding CNS prophylaxis, may be slightly different today. During the study period, few patients were staged using PET-CT and the staging might, therefore, be lower with fewer extranodal involvements in our study. This could have led to an under-estimation of CNS-IPI risk group. Since our results from the Kaplan–Meier analyses are very similar to the results seen in Schmitz et al.^10^ this is however unlikely to be a critical issue.

To summarize, with this national study we provide important benchmark estimates of DLBCL relapse including CNS relapse, quantifying the number of patients that could be candidates for second-line chemotherapy and other new therapies such as with CAR-T cells or bispecific antibodies. We also identify a group of older DLBCL patients unfit for standard immunochemotherapy where new non-chemotherapy-based strategies may be needed already in first-line to help improve outcome.

## Supplementary information

Supplementary legends

Online table 1

Online figure 1

Online figure 2

Online figure 3

Online figure 4

Online figure 5

Online figure 6
