# Supplementary material for: Incidence of relapsed/refractory diffuse large B-cell lymphoma (DLBCL) including CNS relapse in a population-based cohort of 4243 patients in Sweden
Source: Blood Cancer J. 2021 Jan 7;11(1):9. doi: 10.1038/s41408-020-00403-1 (PMC7791057; doi:10.1038/s41408-020-00403-1)
Supplement: Supplementary file 1 — Supplementary legends [file 41408_2020_403_MOESM1_ESM.docx]

**Online table 1.** Treatment for diffuse large B-cell lymphoma (DLBCL) patients considered not curatively treated in this study (n=594) (percentage of the whole cohort of 4243 patients).

**Online figure 1:** Flow chart of data cleaning and exclusions to define the cohort.

**Online figure 2:** Cumulative incidence of relapsed/refractory disease among diffuse large B-cell lymphoma (DLBCL) patients who received 3 or more cycles of chemotherapy (n = 3299, 767 events).

**Online figure 3:** Distribution of relapsed/refractory disease at any location, and of CNS relapse, by time from diagnosis of diffuse large B-cell lymphoma (DLBCL).

**Online figure 4:** Cumulative incidence of relapsed/refractory disease at any site among curatively treated younger patients (<60 years) by aaIPI (0-1, 2-3). (incidence assessed in the presence of the competing risk of death*).

* Not depicted

**Online figure 5:** Cumulative incidence of CNS relapse among patients with CNS-IPI groups 5-6 (n=127, 16 events) (incidence assessed in the presence of the competing risks of relapse without CNS involvement (in light blue) and death (not depicted).

**Online figure 6:** Cumulative incidence of CNS relapse in the absence of competing risks (1 – Kaplan-Meier) by CNS-IPI risk group (detailed level and grouped).
