# Supplementary material for: Incidence of relapsed/refractory diffuse large B-cell lymphoma (DLBCL) including CNS relapse in a population-based cohort of 4243 patients in Sweden
Source: Blood Cancer J. 2021 Jan 7;11(1):9. doi: 10.1038/s41408-020-00403-1 (PMC7791057; doi:10.1038/s41408-020-00403-1)
Supplement: Supplementary file 2 — Online table 1 [file 41408_2020_403_MOESM2_ESM.docx]

**Online table 1.** Treatment for diffuse large B-cell lymphoma (DLBCL) patients considered not curatively treated in this study (n=594) (percentage of the whole cohort of 4243 patients).

| **Treatment** | **N (%)** |
| --- | --- |
| Bendamustine | 17 (0.4) |
| CVP* | 52 (1.2) |
| Gemcitabine | 20 (0.5) |
| Vincristine | 4 (0.1) |
| Other iv chemotherapy | 5 (0.1) |
| Rituximab alone** | 3 (0.1) |
| Only radiotherapy*** | 117 (2.8) |
| Only surgery | 11 (0.3) |
| Oral chemotherapy | 50 (1.2) |
| Best supportive care | 315 (7.4) |

*CVP=cyclophosphamide, vincristine, prednisone. **Some patients may have received rituximab in combination with their chemotherapy regimen. ***Fourteen patients received radiotherapy in combination with chemotherapy.
