# Supplementary figures and images for: Incidence of relapsed/refractory diffuse large B-cell lymphoma (DLBCL) including CNS relapse in a population-based cohort of 4243 patients in Sweden
Source: Blood Cancer J. 2021 Jan 7;11(1):9. doi: 10.1038/s41408-020-00403-1 (PMC7791057; doi:10.1038/s41408-020-00403-1)

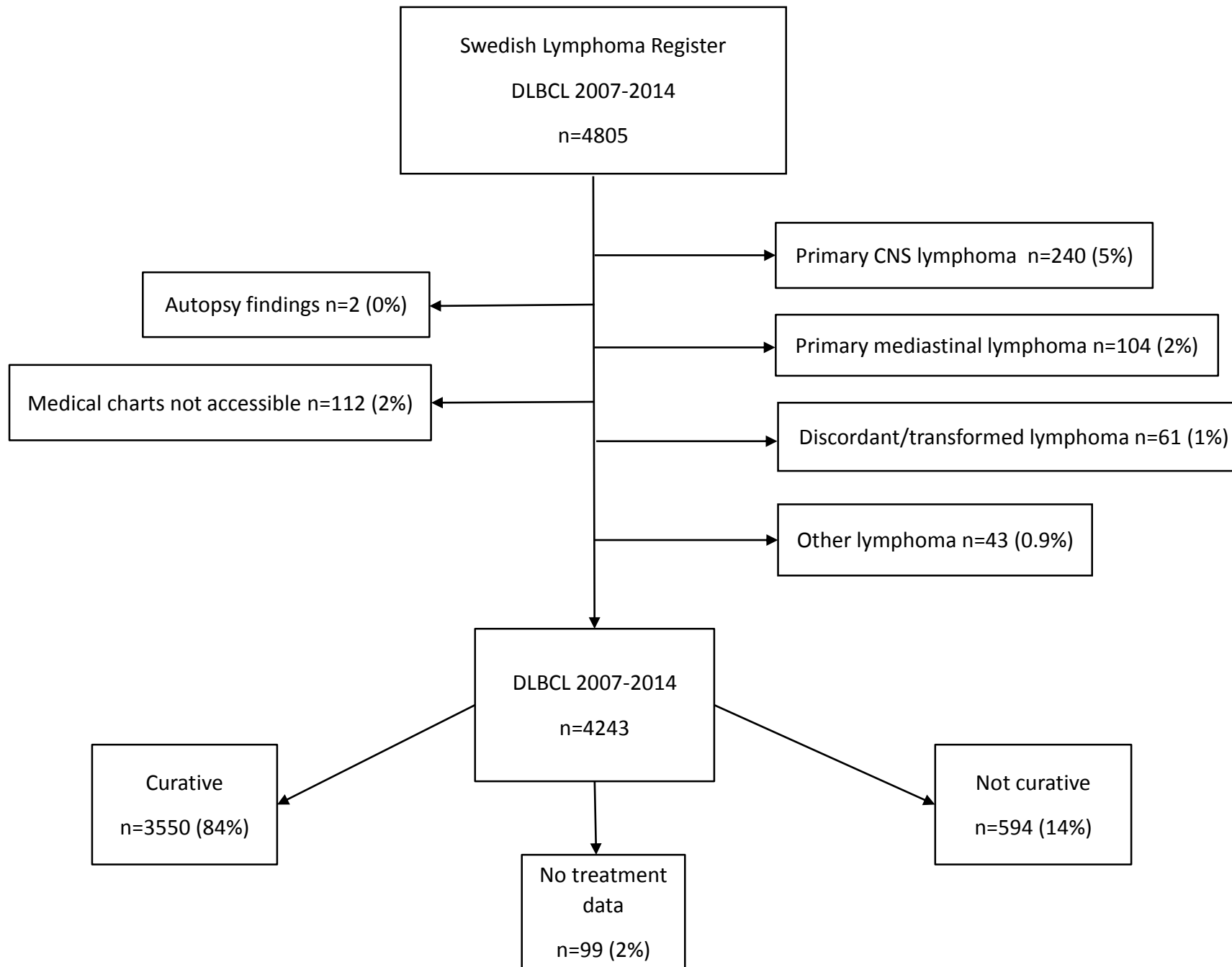

Supplement: Supplementary file 3 — Online figure 1 [file 41408_2020_403_MOESM3_ESM.pdf]

$\geq 3$  cycles of chemotherapy

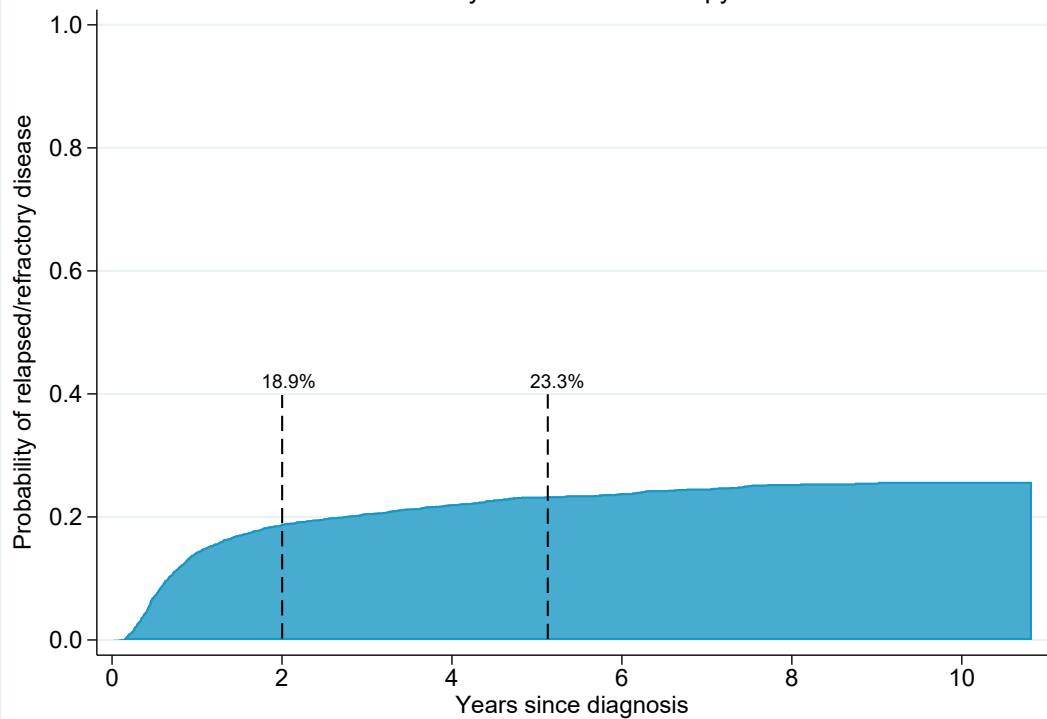

Supplement: Supplementary file 4 — Online figure 2 [file 41408_2020_403_MOESM4_ESM.pdf]

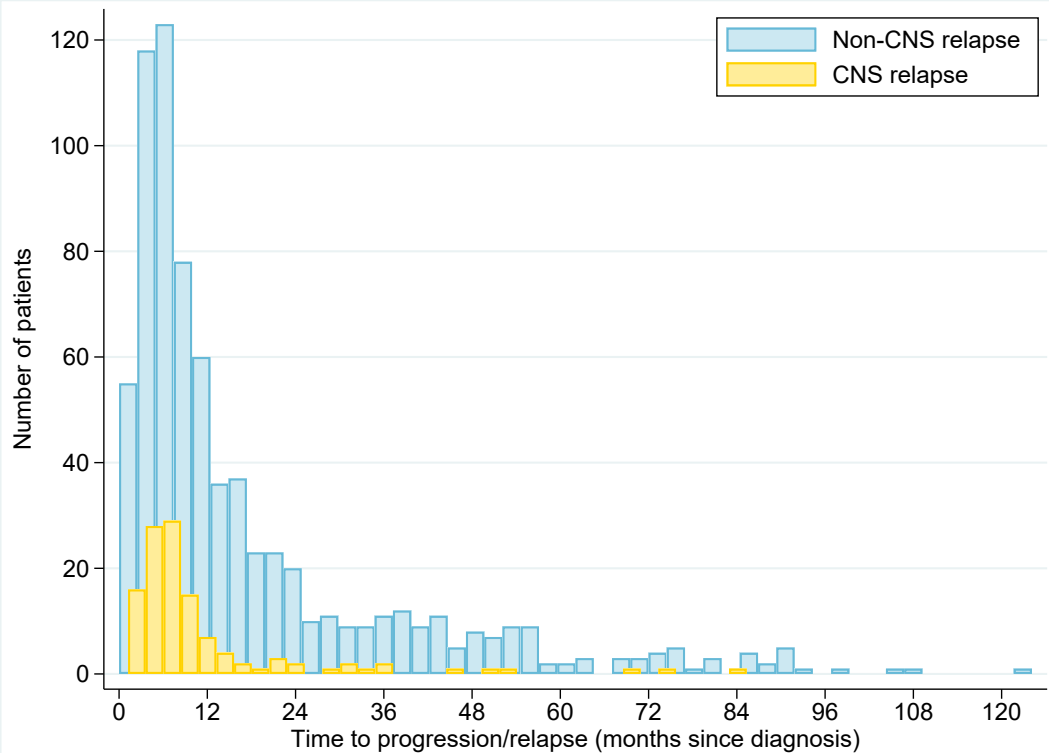

Supplement: Supplementary file 5 — Online figure 3 [file 41408_2020_403_MOESM5_ESM.pdf]

age  $\leq 60$  years  
aalPI: 0,1

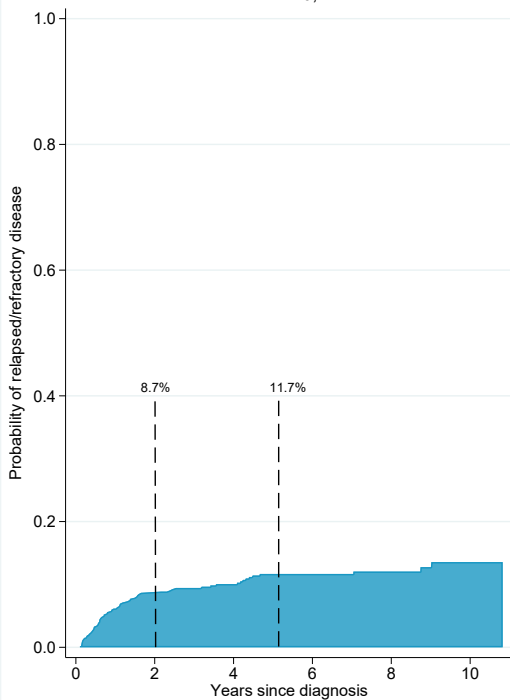

Age  $\leq 60$  years  
aalPI: 2,3

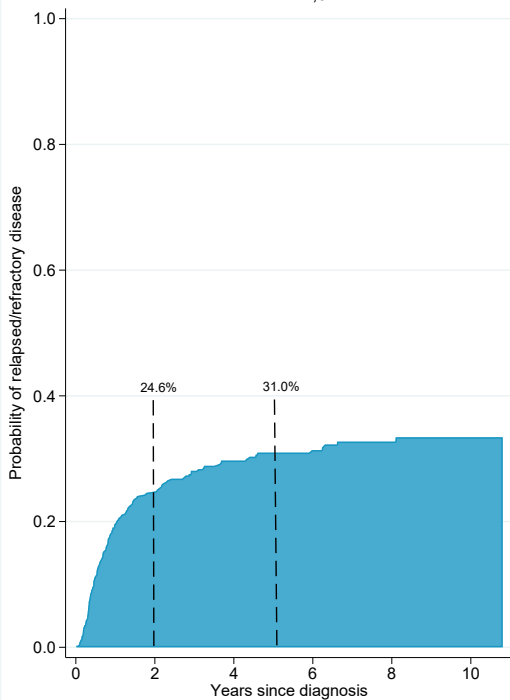

Supplement: Supplementary file 6 — Online figure 4 [file 41408_2020_403_MOESM6_ESM.pdf]

# CNS IPI: 5-6

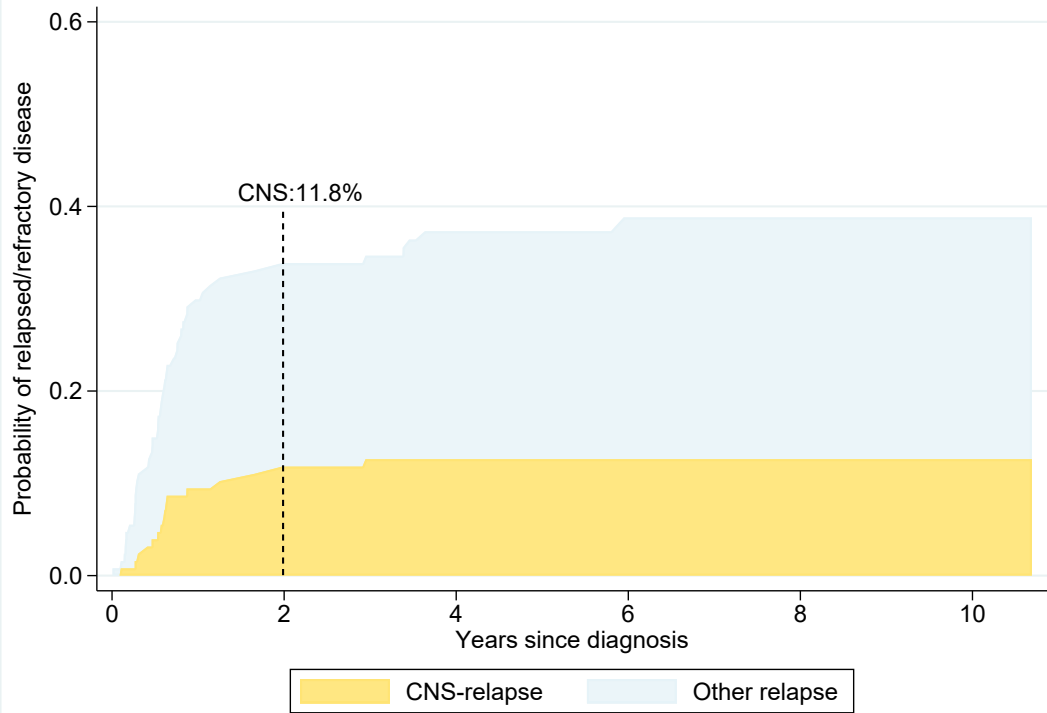

Supplement: Supplementary file 7 — Online figure 5 [file 41408_2020_403_MOESM7_ESM.pdf]

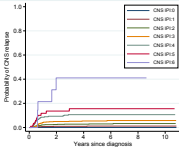

|         |     |      |     |      |     |      |     |      |     |      |    |
|---------|-----|------|-----|------|-----|------|-----|------|-----|------|----|
| At risk |     |      |     |      |     |      |     |      |     |      |    |
| CNS IP0 | 242 | (7)  | 232 | (9)  | 204 | (9)  | 130 | (9)  | 87  | (9)  | 24 |
| CNS IP1 | 777 | (7)  | 858 | (9)  | 817 | (9)  | 318 | (9)  | 184 | (9)  | 38 |
| CNS IP2 | 828 | (21) | 723 | (22) | 528 | (17) | 326 | (17) | 182 | (9)  | 45 |
| CNS IP3 | 828 | (20) | 828 | (22) | 388 | (17) | 227 | (9)  | 118 | (9)  | 28 |
| CNS IP4 | 418 | (25) | 213 | (24) | 158 | (30) | 105 | (30) | 48  | (30) | 15 |
| CNS IP5 | 188 | (10) | 88  | (11) | 35  | (9)  | 18  | (9)  | 18  | (9)  | 2  |
| CNS IP6 | 21  | (8)  | 8   | (9)  | 2   | (9)  | 2   | (9)  | 2   | (9)  | 0  |

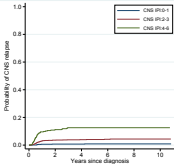

|           |      |      |      |      |     |      |     |      |     |      |    |
|-----------|------|------|------|------|-----|------|-----|------|-----|------|----|
| At risk   |      |      |      |      |     |      |     |      |     |      |    |
| CNS IP0-1 | 1020 | (7)  | 888  | (9)  | 731 | (7)  | 448 | (9)  | 241 | (9)  | 88 |
| CNS IP2-3 | 1775 | (24) | 1220 | (24) | 814 | (24) | 481 | (24) | 277 | (24) | 72 |
| CNS IP4-6 | 848  | (24) | 285  | (24) | 194 | (24) | 120 | (24) | 60  | (24) | 17 |

Supplement: Supplementary file 8 — Online figure 6 [file 41408_2020_403_MOESM8_ESM.pdf]
